# Neuroprotective effects of zonisamide on cerebral ischemia injury via inhibition of neuronal apoptosis

**DOI:** 10.1590/1414-431X202010498

**Published:** 2021-02-26

**Authors:** Junna He, Xiangjian Zhang, Weiliang He, Yanzhao Xie, Yanxia Chen, Yang Yang, Rong Chen

**Affiliations:** 1 Department of Neurology, Second Hospital of Hebei Medical University, Shijiazhuang, Hebei, China; 2 Department of Neurology, Hebei General Hospital, Shijiazhuang, Hebei, China; 3 Department of Endocrinology, Second Hospital of Hebei Medical University, Shijiazhuang, Hebei, China; 4 Hebei Key Laboratory of Vascular Homeostasis, Shijiazhuang, Hebei, China

**Keywords:** Cerebral ischemia, Zonisamide, Neuroprotection, Apoptosis, Neuronal cells

## Abstract

It is known that neuronal apoptosis contributes to pathology of cerebral ischemia injury. Zonisamide (ZNS) has shown anti-apoptosis effects in recent studies. The present study investigated whether the anti-apoptotic effect can account for the neuroprotective action of ZNS on cerebral ischemia. Neuronal cells were maintained under oxygen-glucose deprivation conditions to simulate cerebral ischemia and treated with ZNS simultaneously. The apoptosis of the cells and expression of apoptosis-related proteins were investigated by flow cytometry and western blot analysis, respectively. A cerebral ischemia mouse model was created via middle cerebral artery occlusion, and the mice were treated with ZNS. Neurological deficit scores and infarct volumes of the cerebral ischemia mice were measured. The apoptosis status of the neuronal cells was evaluated by TUNEL staining. *In vitro*, the ZNS treatment inhibited both the apoptosis of the neuronal cells and apoptosis-related protein expression (caspase-3, caspase-8, and calpain-1) induced by the oxygen-glucose deprivation. The anti-apoptosis effect of ZNS could occur through the blocking of reactive oxygen species. Moreover, ZNS treatment significantly ameliorated neurological deficits and reduced infarct volumes in the cerebral ischemia mice model. In this study, ZNS exerted neuroprotective effects by inhibition of apoptosis in neuronal cells in cerebral ischemia. Therefore, ZNS might be a promising therapy for cerebral ischemia.

## Introduction

Cerebrovascular accidents (stroke), including cerebral ischemia, are the second leading cause of death and the third leading cause of disability globally (1). Cerebral ischemia leads to irreversible pathological injury in the penumbra, such as energy failure, ionic homeostasis loss, excitotoxicity, increased oxidative stress, and cell apoptosis, resulting in neuronal necrosis (2). In recent years, although much progress has been made in the treatment of cerebral ischemia, such as antithrombotic therapy and neuroprotective therapy (3), efficient therapeutic strategies to minimize the damage to neurons and assist functional recovery are still lacking. Typically, apoptosis is considered the main cause of neuronal death (4), and some studies have identified the activation of apoptosis in the penumbral region (5). Hence, inhibition of apoptosis during cerebral ischemia may be an ideal option to salvage the neurons of the penumbra and ensure their survival. In certain preliminary studies, anti-apoptosis treatment was shown to improve the prognosis of cerebral ischemia (6).

Research has also shown that reactive oxygen species (ROS) participate in the pathology of brain injury after cerebral ischemia. Under physiological conditions, ROS play important roles in signaling and metabolic pathways. However, tissue injury after cerebral ischemia would increase levels of ROS, and then excess ROS could induce destruction of cellular proteins, lipids, and DNA, and could trigger apoptosis of neuronal cells (7,8). For example, ROS can increase the permeability of the mitochondrial membrane and the release of cytochrome c, activate caspase, and finally activate apoptosis. Excitotoxicity is another mechanism of neuronal injury following cerebral ischemia. Through NMDA receptors, excitotoxicity induces an influx of calcium ions and then promotes neuronal death (9,10). Moreover, increased mitochondrial calcium ion overload by excitotoxicity could contribute to the generation of superoxide and may promote the expression of pro-apoptotic proteins (11).

Zonisamide (ZNS) is known as an anticonvulsant drug, and it acts as an inhibitor of voltage-dependent Na^+^ and T-type Ca^2+^ channels, which decreases the likelihood of action potential generation (12). ZNS exhibits certain anti-apoptosis effects on neurons. In a study on dopaminergic neurons, Condello et al. (13) found that ZNS preserves mitochondrial functions and counteracts apoptotic signaling in cells. Therefore, we speculated that ZNS might contribute to the survival of neurons in the pathology of cerebral ischemia. In the present study, we investigated the anti-apoptosis effect of ZNS in the neurons. Our data showed that ZNS inhibited the apoptosis of neurons by an antioxidant action *in vitro*. Moreover, ZNS can decrease the area affected by cerebral ischemia by blocking apoptosis in a mouse model with cerebral ischemia.

## Material and Methods

### Experimental mouse model

All animal care procedures and experiments were conducted according to the “ARRIVE” guidelines and approved by the Committee of Ethics on Animal Experiments of Hebei Medical University (No. 1409045). Male C57BL/6 mice (25-30 g, 10 weeks old) were obtained from Vital River Laboratory Animal Technology Co., Ltd. (China). All the mice were bred and housed with food and water available *ad libitum* under standard conditions (12-h light/dark cycle, humidity: 60±5%, and temperature: 22±3°C).

Cerebral ischemia was induced by permanent middle cerebral artery occlusion (MCAO) via insertion of surgical filaments (Beijing Sunbio Biotech Co., Ltd., China) (14,15). In brief, the mice were anesthetized with an intraperitoneal injection of 4% chloral hydrate (350 mg/kg). A midline incision was made on the ventral side of the neck to expose the common carotid arteries. Thereafter, the external carotid artery (ECA) was ligated at two locations using surgical nylon monofilaments near the bifurcation of the ECA and internal carotid artery (ICA). Then, an incision was made between the two ECA ligatures, and a nylon filament (diameter: 0.25±0.03 mm, length: 2 cm) was gently inserted into the lumen of the ICA through the incision. The sham-operated mice underwent the same operation procedure without the insertion of the nylon filament. Twenty-four hours after the experiment, the mice were euthanized via CO_2_ inhalation (30% of the chamber volume per min), and their brain tissues were collected.

The mice were randomly allocated to three groups. In the ZNS group, the mice received the MCAO operation and were treated with ZNS (30 mg/kg) intraperitoneally 1 h after ischemia. In the MCAO group, the mice received the MCAO operation and were treated with saline (30 mg/kg) intraperitoneally 1 h after ischemia. In the sham group, the mice underwent a sham operation and received saline (30 mg/kg) intraperitoneally 1 h after ischemia.

ZNS was purchased from Dalian Mellon Biotech Co. Ltd. (China) and was dissolved in saline at a concentration of 30 mg/mL for the experiments (16).

### Cell culture

HT22 cells were purchased from the Institute of Biochemistry and Cell Biology, Chinese Academy of Sciences, and maintained in DMEM (Sigma, USA) supplemented with ultracentrifuged FBS (Invitrogen, USA) at 37°C in a humidified atmosphere of 5% CO_2_. The cells were treated with 50 μM ZNS (dissolved in saline) (17) or 1 mM H_2_O_2_ (18).

The cells were divided into six treatment groups: 1) oxygen glucose deprivation (OGD) treatment to simulate cerebral ischemia *in vitro*. The HT22 cells were maintained in a serum-free and sugar-free medium in a chamber with 5% CO_2_ and 95% N_2_ at 37°C for 2 h; 2) OGD + ZNS group, the cells were maintained in a serum-free and sugar-free medium containing 50 μM ZNS in a chamber with 5% CO_2_ and 95% N_2_ at 37°C for 2 h; 3) H_2_O_2_ group, the cells were cultured in a regular medium containing 1 mM H_2_O_2_ in a regular incubator with humidified 5% CO_2_ at 37°C for 24 h; and 4) ZNS + H_2_O_2_ group, the cells were cultured in a regular medium containing 1 mM H_2_O_2_ and 50 μM ZNS in a regular incubator with humidified 5% CO_2_ at 37°C for 24 h. The control groups comprised cells cultured in a regular medium in a regular incubator with humidified 5% CO_2_ at 37°C for 2 h (group 5) or 24 h (group 6).

### Neurological deficit scores

A neurological test was performed by an investigator blinded to the experiment. Neurological deficit was scored based on the below-mentioned features, according to a previous study (19): 0) no deficits; 1) torso flexion to the contralateral side; 2) spontaneous circling to the contralateral side; 3) falling to the contralateral side; and 4) no spontaneous movement or unconsciousness.

### Cerebral infarct volumes

The brain tissues were collected after euthanasia, and then frozen in liquid nitrogen, and stored at -80°C. Thereafter, the brain tissues were serially sliced to 20-μm thick coronal sections on a cryostat every 400 μm. The slices were stained with 1% 2,3,5-triphenyl tetrazolium chloride at 37°C for 20 min followed by fixing with 4% paraformaldehyde overnight at room temperature. All the stained sections were digitally photographed, and the images were analyzed by ImageJ software (NIH, USA). Infarct volumes were calculated using the following formula: percentage hemisphere lesion volume (%HLV) = [total infarct volume − (volume of intact ipsilateral hemisphere − volume of intact contralateral hemisphere)] / contralateral hemisphere volume × 100% (20).

### TUNEL staining

Paraffin-embedded 5-µm thick coronal brain sections were dewaxed, and the antigen retrieval procedure was performed. Then, the slides were incubated with a mixture of TdT and biotin-dUTP reagent provided in the TUNEL assay kit (Roche, USA) according to the manufacturer's protocol, followed by staining with streptavidin-HRP and DAB substrate. TUNEL-positive cells were counted in three non-overlapping visual fields of the ischemic cortex region selected around the infarct core for each section under a 400× light microscope (Olympus, Japan).

### Cell apoptosis assay

Flow cytometry was applied to evaluate the apoptosis of the cells *in vitro*. The HT22 cells from the different pretreatments were collected and washed with PBS to remove debris. Thereafter, the cells were resuspended and incubated in buffer (500 μL) containing propidium iodide (PI, 10 μL) and annexin V-FITC (5 μL) purchased from Beyotime (China) for 30 min at room temperature in a dark environment. The apoptosis ratio of the cells was measured by a FACScan flow cytometer (Becton Dickinson, USA) according to the instructions.

### Western blot

Protein extraction from the cell pellets was performed using a total protein extraction kit (Applygen Technologies, China) in accordance with the manufacturer's protocol. The protein concentrations were determined using a BCA protein assay reagent kit (Novagen, USA). Then, 20 μg of total protein samples were loaded and separated by SDS/PAGE electrophoresis and transferred to PVDF membranes (Millipore Corporation, USA). After blocking with 5% skim milk in TBST buffer (Tris-buffered saline, 0.1% Tween 20) at room temperature for 2 h, the membranes were incubated with the primary antibody (1:5000) at 4°C for 8 h. Subsequently, the membranes were incubated with a secondary antibody (1:5000, anti-rabbit, or anti-mouse IgG, Abcam, USA). Then, the protein level on the blot was detected using the Western Bright ECL kit (Bio-Rad Laboratories, USA). The following antibodies against the target proteins were used: rabbit monoclonal anti-calpain-1 antibody (ab108400, Abcam), rabbit monoclonal anti-caspase-3 antibody (9664, Cell Signaling Technology, USA), mouse monoclonal anti-caspase-8 antibody (9496, Cell Signaling Technology), and rabbit polyclonal anti-GAPDH antibody (ab9485, Abcam). Detection of GAPDH was used as the internal control.

### Reactive oxygen species assay

We used Reactive Oxygen Species Assay kit (Beyotime) to investigate ROS generation by tissues. In brief, homogenates were diluted in Locke's buffer at a concentration of 10 mg tissue/mL. The homogenates were added into 24-well plates (0.45 mL/well), and then incubated with 5 μL DCFH-DA (10 mM) at room temperature for 30 min. Hereafter, 50 μL Fe^2+^ was added to the wells and incubated for 30 min. Finally, the fluorescent activity was measured by a multiple plate reader (Thermo Fisher Scientific, USA) with excitation at 485 nm and emission at 530 nm.

### Statistical analysis

All the data were analyzed in an R environment for statistical computing and graphics (version 3.6.2). All the *in vitro* experiments were triplicated. The quantitative data are reported as means±SE. Statistical comparisons were performed by one-way ANOVA followed by the Tukey *post-hoc* test. Neurological deficit assessment was tested by applying the Student's *t*-test for comparisons between two groups. Differences with P<0.05 were considered to be significant.

## Results

### ZNS inhibited apoptosis of neuronal cells subjected to OGD

**Figure 1:**
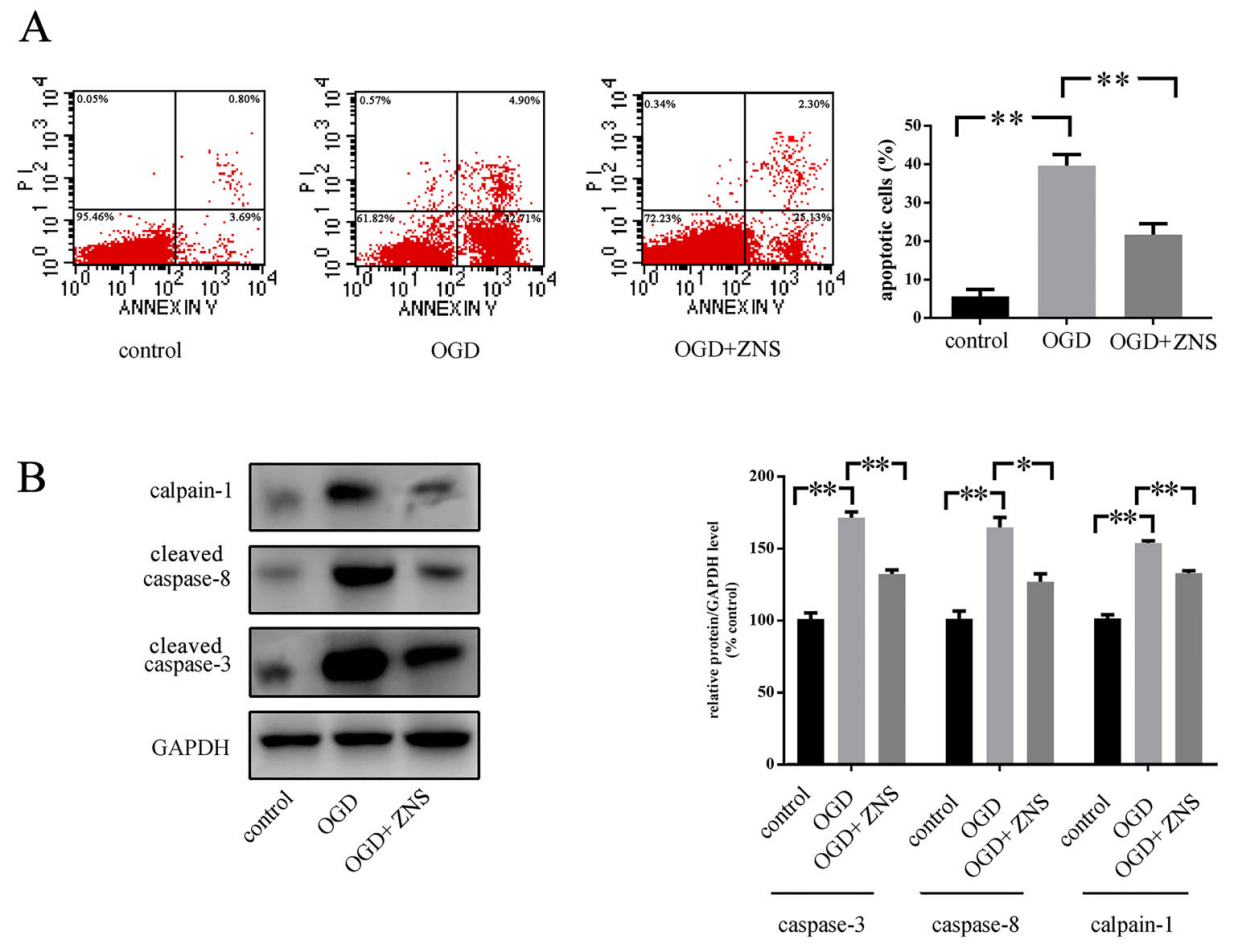
HT22 cells were pretreated or not with zonisamide (ZNS), and then exposed to oxygen glucose deprivation (OGD) conditions for 2 h. **A**, The number of apoptotic (annexin V-positive) cells is indicated as the percentage of gated cells. Representative images and relative quantifications are shown. **B**, Apoptosis-related protein expression in cells under OGD. All the experiments were performed in triplicate, and the data are reported as means±SE (*P<0.05, **P<0.01 one-way ANOVA).

A large number of apoptotic cells were clearly observable after OGD treatment compared to that without OGD treatment (5.45% in control, 39.67% in OGD, 21.38% in OGD+ZNS, Figure 1A). In addition, all the apoptosis-related proteins (caspase-3, caspase-8, and calpain-1) showed an increase in the cells subjected to OGD (Figure 1B). However, co-treatment by ZNS significantly inhibited the apoptosis rate of cells under OGD (Figure 1A), and accompanied decreased expressions of caspase-3, caspase-8, and calpain-1 (Figure 1B).

**Figure 2:**
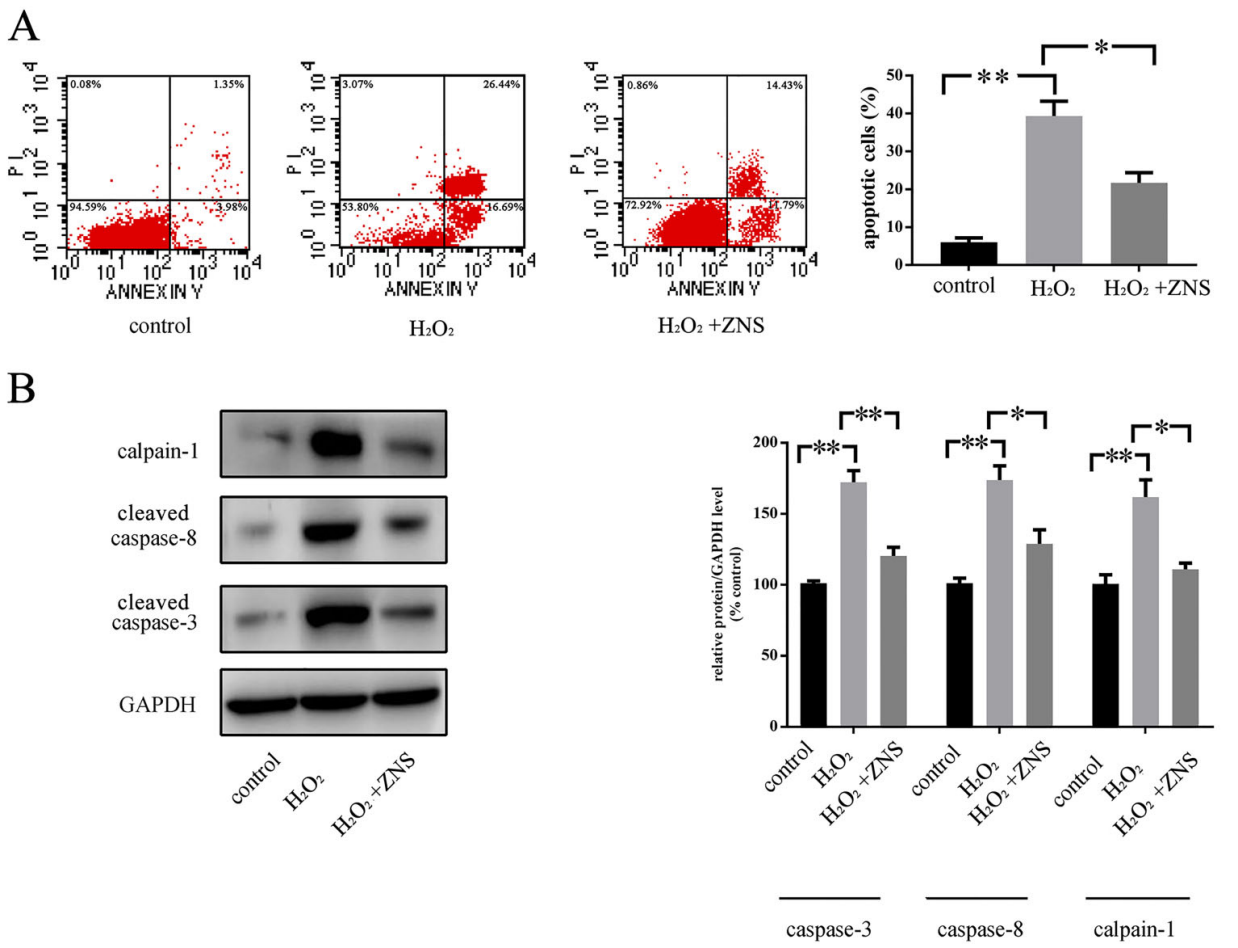
The cells received zonisamide (ZNS) or H_2_O_2_, for 24 h. **A**, Cells stained with annexin V and PI, showing the number of apoptotic (annexin V-positive) cells as the percentage of gated cells. **B**, Apoptosis-related protein expression in cells after different treatments via western blot (representative images and relative quantifications are shown). All the experiments were performed in triplicate, and the data are reported as means±SE (*P<0.05, **P<0.01 one-way ANOVA).

**Figure 3:**
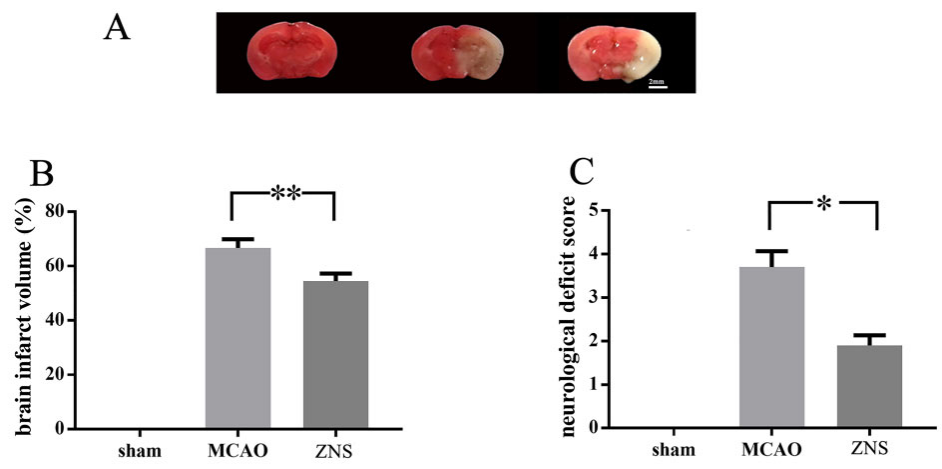
Effect of zonisamide (ZNS) on neurological deficit and infarct size in middle cerebral artery occlusion (MCAO) mice. **A**, Representative images of brain sections (from left to right: sham, MCAO, and ZNS; scale bar: 2 mm) and (**B**) statistical results. **C**, Mice with ZNS treatment showed lower neurological deficit scores. ZNS: middle cerebral artery occlusion surgery + ZNS treatment; sham: sham surgery. The data are reported as means±SE (*P<0.05, **P<0.01 one-way ANOVA).

**Figure 4:**
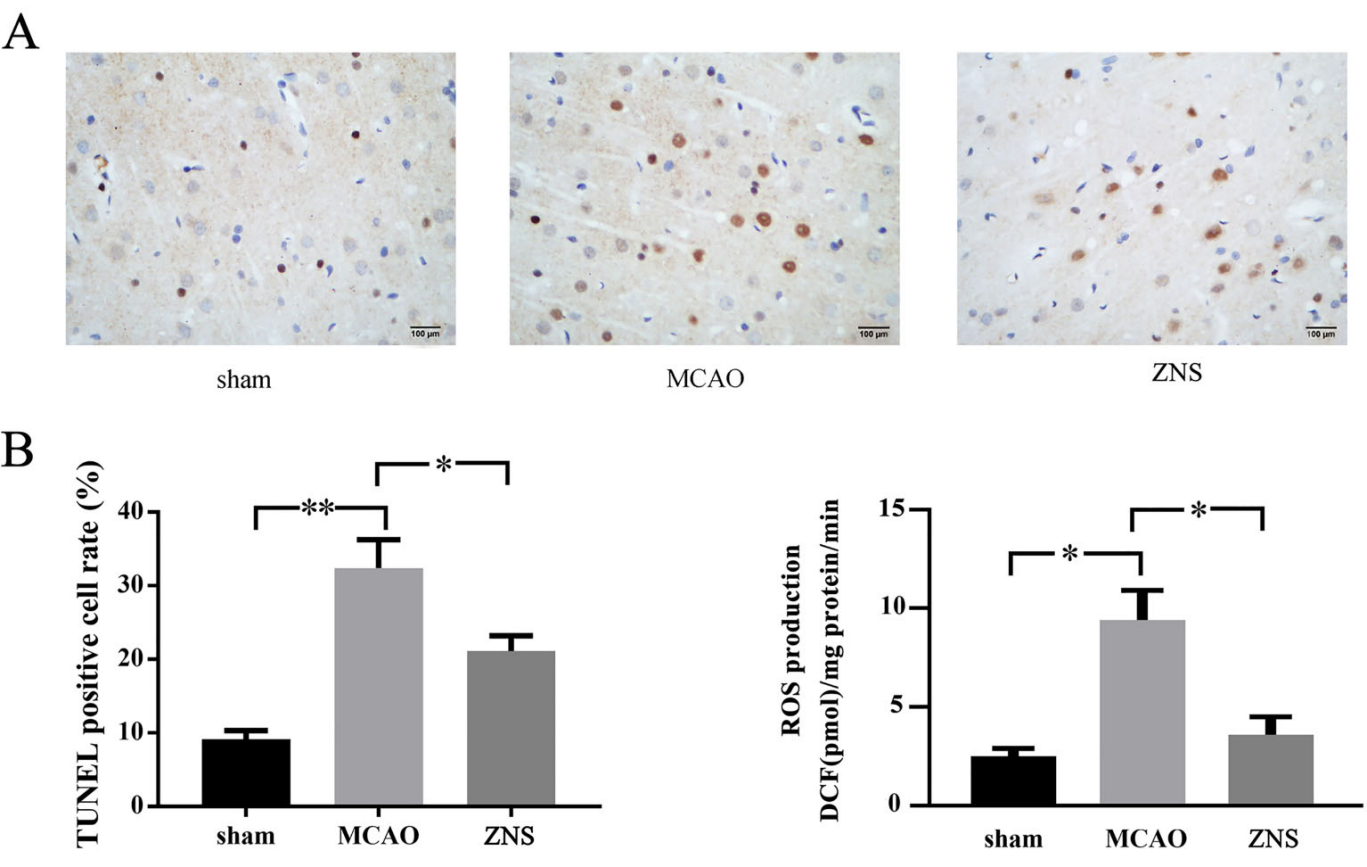
Treatment with zonisamide (ZNS) inhibits apoptosis of neuronal cells and reactive oxygen species (ROS) production in the penumbra of middle cerebral artery occlusion (MCAO) mice. **A**, Representative images of TUNEL staining (400×, scale bar: 100 μm). **B**, Mice with ZNS treatment showed fewer TUNEL-positive cells in the brain slices and lower reactive oxygen species (ROS) levels, measured in DCF fluorescence, than the group without ZNS treatment. ZNS: middle cerebral artery occlusion surgery + ZNS treatment; sham: sham surgery. The data are reported as means±SE (*P<0.05, **P<0.01 one-way ANOVA).

### ZNS blocked apoptosis of neuronal cells by blocking the effect of ROS

The apoptosis rate increased after H_2_O_2_ treatment, whereas the combination treatment with ZNS alleviated the pro-apoptosis effect of H_2_O_2_ (6.13% in control, 39.33% in H_2_O_2_, 21.67% in H_2_O_2_+ZNS). Additionally, ZNS decreased the expression of pro-apoptosis proteins (caspase-3, caspase-8, and calpain-1) induced by H_2_O_2_ (Figure 2).

### ZNS reduced the neurological deficit score and infarct size after cerebral ischemia

Neurological deficit was investigated at 24 h after surgery. Cerebral ischemia developed in the mice after MCAO surgery. Compared to the control group that received sham surgery, both the neurological deficit and the cerebral infarct area were observed in the MACO mice group (Figure 3). Moreover, our results showed that treatment with ZNS significantly improved the cerebral ischemia outcome, as lower neurological deficit scores (3.55 in MCAO *vs* 1.99 in ZNS) and lower infarct volumes (67.93% in MCAO *vs* 54.48% in ZNS) were recorded.

### ZNS attenuated apoptosis and generation of ROS after cerebral ischemia

The results of TUNEL staining showed apoptosis of mice brain neuronal cells after MCAO surgery (Figure 4A). As shown in Figure 4B, the number of TUNEL-positive cells in the brain slices of mice in the ZNS group were less than those in the group without ZNS treatment (9.14% in Sham, 32.38% in MCAO, 21.12% in ZNS). Furthermore, significantly decreased ROS levels were found in the brain tissues of mice in the ZNS group, compared to those in the group without ZNS treatment (Figure 4B)

## Discussion

In this study, we investigated the effect of ZNS on the neuronal cells of mice with cerebral ischemia *in vitro* and *in vivo*. Our results showed that cerebral ischemia induced apoptosis of neuronal cells, while ZNS treatment inhibited it by blocking the effect of ROS. Moreover, ZNS treatment improved cerebral ischemia outcomes, as evidenced by the lower neurological deficit scores and lower infarct volumes.

Under cerebral ischemia, the generation of ROS in cells is increased, overwhelming the antioxidant systems, which include stable oxidants, such as H_2_O_2_, and unstable free radicals, such as superoxide anion, nitric oxide, hydroxyl moiety, and hypochlorite. Excess ROS generation can induce the oxidation of DNA, lipids, and peptides of cells, which would damage the cells and induce tissue dysfunction. Mitochondria could be the primary source of ROS involved in ischemia-induced apoptosis. Mitochondrial ROS influence the release of cytochrome c and other apoptotic proteins (such as caspase-3, caspase-8, and calpain-1) from the mitochondria into the neuronal cytosol, which leads to apoptosis after stroke (8,21). Hence, the generation of ROS could induce cell damage and changes of pathways to contribute to the cell death after cerebral ischemia. In addition, excess ROS generation can induce the progression of inflammatory disorders, and then cause tissue injury and dysfunction of vascular endothelium. In contrast, some anti-ROS reagents improve the treatment outcomes of stroke through the inhibition of inflammation. In a rat model of cerebral ischemia, Lopes et al. (22) found that anti-inflammatory indomethacin modulates microglia activation and promotes proliferation and migration of neuroblasts. Moreover, Costa et al. (23) reported that astragaloside enhances function recovery of patients with cerebral ischemia due to its antioxidant, anti-apoptotic, and anti-inflammatory properties.

Some previous studies have addressed the neuroprotective effect of ZNS. ZNS is normally indicated in therapy for partial seizures in adults with epilepsy. ZNS blocks both Na^+^ and T-type Ca^2+^ channels, hyperpolarizes neuronal membrane potential, reduces spontaneous firing, and inhibits neuronal excitability (24). Furthermore, ZNS has other pharmacological effects, including an anti-oxidant effect (25), and increasing expression of brain-derived neurotrophic factor, nerve growth factor, and neurotrophic receptor tyrosine kinase 2 (26). Our study showed the neuroprotection against cerebral ischemia, which is consistent with the study by Minato et al. (27). Moreover, our results also showed that treatment with H_2_O_2_ increased the apoptosis of HT22 cells, while pretreatment with ZNS could attenuate the pro-apoptosis effect of H_2_O_2_ on the neuronal cells. We suggest that this anti-apoptosis effect of ZNS was due to the enhanced free radical scavenging capability. Kawajiri et al. (17) reported that ZNS increases the level of superoxide dismutase-2 in the SH-SY5Y cells, thus preventing cell apoptosis. Moreover, some recent studies demonstrate the neuroprotective effect of ZNS against oxidative stress-induced neurotoxicity on dopaminergic neurons in mice (28,29). Besides the effects on anti-ROS, the protective role of ZNS in the pathology of cerebral ischemia could be attributed to some other pathways involving the ERK1/2 pathway. Multiple studies have demonstrated that ERK1/2 phosphorylation plays an active role in mediating H_2_O_2_-induced apoptosis of cells (30–32). Yagi et al. (26) reported that H_2_O_2_ promotes phosphorylation of ERK1/2 in primary motor neurons, which can be attenuated by treatment of ZNS.

Neurons are one of the cell types most susceptible to ischemic injury. Under cerebral ischemia, neuronal cells experience OGD, which in turn leads to hypoxic conditions and energy depletion. Because the neurons have a high energy demand, loss of energy and oxygen lead to cell apoptosis and death, finally inducing irreversible neurodegeneration. Multiple studies have shown that acute ischemic cerebral ischemia activates the apoptotic pathways of neuronal cells (33,34). The present study also showed that blocked blood supply of ICA induced cerebral ischemia insults and induced apoptosis of neuronal cells. Hence, researchers and clinicians believe that anti-apoptosis therapy may help the neuronal cells survive during cerebral ischemia and improve treatment outcomes. Some preclinical studies have provided evidence that anti-apoptosis treatment may limit neuronal injury in stroke. Zheng et al. (35) reported that catalpol exerts neuroprotective effects by inhibiting apoptosis in mice with acute focal ischemic stroke and improved neurological function recovery. Liu et al. (36) reported that kukoamine A could alleviate ischemia injury by inhibition of expressions of caspase-3 and cytochrome c, and the ratio of Bax/Bcl-2. The results of this study indicated that anti-apoptosis might be a promising adjustment treatment in cerebral ischemia, although we have to admit that to date, this anti-apoptosis treatment is still preliminary, and more research is needed to validate the outcome and develop a protocol for anti-apoptosis treatment. Furthermore, some doubts regarding anti-apoptosis treatment in cerebral ischemia persist. For example, some researchers question the clinical effectiveness of anti-apoptosis treatment, since direct evidence to demonstrate that neuronal apoptosis contributes to the deterioration of neurological function is still lacking. Moreover, it is unclear whether anti-apoptosis treatment ultimately improves the function of the central nervous system. The side effects of anti-apoptosis treatment also remain unknown. A study on brain development suggests that apoptosis plays an important role in the developing nervous system (37). Therefore, more research is needed to explore the effect of apoptosis in the pathophysiology of cerebral ischemia.

This study has some limitations. We only investigated the effect of ZNS in neuronal cells, whereas multiple types of cells exist in the central nervous system (e.g., astrocytes, microglial cells, ependymal cells, and oligodendrocytes). Further research needs to be conducted to determine whether ZNS has an anti-apoptosis effect on other types of cells in the central nervous system. In our study, cerebral ischemia was induced by permanent MCAO via the insertion of surgical filaments, and the mice were euthanatized 24 h after surgery. However, these conditions cannot fully simulate the pathology of cerebral ischemia in humans. An animal model that most closely simulates human ischemic stroke pathology needs to be developed to help researchers assess the long-term effects of ZNS on cerebral ischemia.

### Conclusions

Our study showed that ZNS inhibited the apoptosis of neuronal cells in cerebral ischemia by the blocking the effect of ROS. Moreover, ZNS treatment improved the outcome of cerebral ischemia, and thus, it might be a promising therapeutic strategy for cerebral stroke.
